# Exocortex Network for AI-Augmented Human-Led Scientific Expedition

**DOI:** 10.1021/photonsci.5c00009

**Published:** 2025-10-22

**Authors:** Esther H. R. Tsai, Kevin G. Yager

**Affiliations:** Center for Functional Nanomaterials, 8099 Brookhaven National Laboratory, Upton, New York 11973, United States

**Keywords:** artificial intelligence, machine-learning, foundation models, human-computer interaction, exocortex

## Abstract

AI advances in science can be viewed along two main directions with a fluid boundary: enhancing efficiency through automation and smart tools to accelerate tasks that humans can already perform; and enabling exploration into uncharted territories and potentially toward AGI. These advances manifest in the AI cognitive core through the development and explainability of foundation models; in the physical embodiment of instruments and facilities; and in the integrated agency of AI workflows exemplified by the science exocortex. To address the role of humans in this evolving landscape, in this Perspective, we suggest a third direction: the development of personalized agents that form human-centered networks, supporting both efficiency and exploration while ensuring that AI remains aligned with human vision.

## Graphical abstract

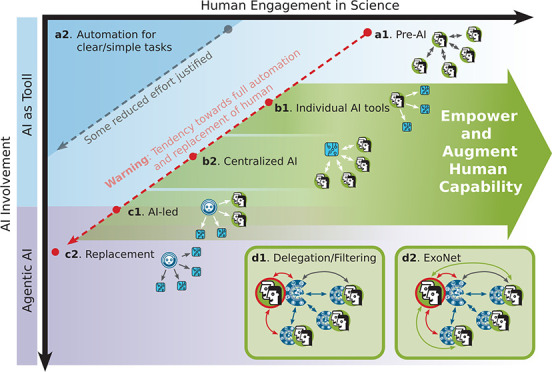

## Introduction

Over recent years, automation methods, often leveraging artificial intelligence and machine-learning (AI/ML) have gradually redefined the fundamental processes and methodologies for scientific research and, in some cases, accelerated discovery.^1–4^ Automation is not merely a convenience; many problem spaces are not amenable to search manually. For instance, materials discovery often involves exploration of high-dimensional spaces, making it infeasible to exhaustively search for optimal structures or properties. With increasing automation adoption across different areas, including hardware robotics and analysis software, more laboratories are moving toward “closed-loop” experimental workflows. For example, samples are prepared using robotic or combinatorial methods, and then key measurements are taken and data analyzed. The resulting data are fed into a decision-making method that selects high-value follow-up samples to synthesize/measure according to user-defined criteria. This creates a continuous cycle of measurement, analysis, and adjustment, and hence, the term “closed-loop”. Self-driving lab (SDL)^5–7^ and autonomous experimentation (AE)^8–11^ can refer to variations of this framework, some focusing more on automation with robotics^12^ and some on developing tailored decision-making methods.^9^ For decision-making, Gaussian processes serve as a powerful probabilistic approach within the Bayesian optimization framework, with applications in material synthesis,^13–15^ material processing,^16,17^ instrumentation configuration,^18^ and characterization via diffraction^19–21^ and microscopy.^22,23^ While automation of routine tasks can reduce human effort, the integration of advanced AI, whether autonomous methods or agentic AI, should serve to augment scientists’ capabilities and actually promote human engagement in science.

With the emergence of foundation models,^24^ in particular, large language models (LLMs),^25,26^ science is on the cusp of another major transformation.^27–31^ Unlike earlier AI systems, which are often task-specific or narrow in scope, these newer models bring much broader capabilities, including understanding complex natural language expression,^32,33^ efficient information retrieval,^34,35^ reasoning^36–39^ and hypotheses generation,^38–41^ and multi-agent orchestration.^42–50^ LLM applications have been demonstrated in material science and chemistry,^51,52^ including molecular and material design,^53–61^ experimentation,^62–69^ data captioning,^70^ benchmarking,^71–74^ and multi-agent workflow.^75^ Foundation models and applications open the door to AI-augmented scientific expeditions, enabling researchers to venture into uncharted intellectual territory and pursue discoveries that were previously unimaginable due to time, scale, or complexity constraints. By serving as collaborators rather than just tools, LLMs can help scientists explore questions they might not have considered, identify hidden connections across disciplines, and navigate a vast knowledge base and ideas. Just as automation revolutionized the “doing” of science, LLMs and foundation models may revolutionize the “thinking” of science via empowering humans with enhanced capabilities and fresh perspectives.

Imagine a near future, where every scientist has an exocortex^76^ that acts as an external layer of cognition to augment a scientist’s abilities. The swarm of AI agents that constituted the personal exocortex can provide guidance on the scientific instrument operation,^64^ correlate multi-modal data to provide new insights, connect simulations with multiple experimental results for holistic studies, and search the literature^77^ to propose new theoretical or experimental studies. The exocortex is a broad and ambitious vision for the future of science, which will require advancements in AI/ML methods and tailoring of these advancements to science workflows. Nevertheless, we can see early hints of such agentic and multi-agent approaches accelerating research.^78–80^ As a concrete example, we have been developing a scientific companion^64,67^ that leverages a designed sequence of LLM calls to behave as a natural-language interface to complex instrumentation at scientific user facilities. The system is intended to alleviate the burden for instrument scientists (training, troubleshooting, etc.) and increase engagement from users (as it allows them to more rapidly become independent operators), thereby expanding the complexity of possible experiments. Using this approach, we recently demonstrated the first voice-controlled experiment^64^ at a synchrotron^81^ beamline that hosts complex experiments, contributing one component of the exocortex.

While the exocortex (exo) concept is not yet realized, it is not too soon to begin imagining further improvements to the concept. In particular, the exocortex is envisioned foremost as a means of expanding the cognition and volition of an individual scientist, allowing them to orchestrate a diverse catalogue of science resources (publications, databases, software, instruments, etc.). Yet we must acknowledge that modern science is fundamentally a community activity. The reasons for this are many: the key role of peer-review as a self-correction mechanism, the improved creativity arising from group brainstorming, the growing need for interdisciplinary approaches to tackle the grandest challenges, and perhaps most importantly—because humans are social creatures that thrive when part of a community. In this context, here, we propose the concept of a scientific exocortex network (ExoNet) to connect not only human-to-AI, but also human-to-human, to efficiently create productive human partnerships that consistently drive human-led science. We envision a world where all our exocortices are connected, continuously facilitating collective brainstorming, ideation, and, most importantly, forming dynamic communities (sub-nets) that connect human scientists with shared passion and vision. Based on our experience of natural-language-based interactions at synchrotron beamlines,^64^ in this Perspective, we continue to use synchrotron X-ray experiments as illustrative examples to discuss the envisioned impact of ExoNet, while noting that its potential influence extends broadly to other facility instruments and across the scientific community.

## Exocortex Network

Humans are driven by aspiration and creativity, yet the demands of daily life often constrain us to routine, repetitive tasks, limiting the time and cognitive resources available for higher-order thinking and innovation. The fundamental question is how can AI be leveraged to alleviate these burdens and free human capacity for creative and impactful work? Recent advances in LLMs are enabling AI to manage many well-defined tasks, while human scientists may tackle complex challenges and develop long-term vision. However, a chasm between technological advances and scientific experimentation, coupled with a lack of common language and shared interests across disciplines, often results in sporadic and disorganized testing of AI/ML concepts in physical science communities. We are far from realizing the full potential of the current AI methods for improving science. Looking further into the future, one can extrapolate the rapid progress in AI capabilities^82,83^ to predict the upcoming development of human-level artificial general intelligence (AGI).^84–86^ The potential of AGI engenders a host of concerns, as such powerful systems would be difficult to control and by default may well displace humans instead of empowering them. While much of the current focus in agentic AI centers on replicating human abilities and thereby replacing human workers,^87^ we argue that the true opportunity lies in leveraging frontier AI to enhance human creativity, broaden knowledge, foster efficient cross-disciplinary collaboration, and achieve greater productivity. Therefore, in this Perspective, we suggest a human-centered agentic AI framework where a human sets the goal, and AI agents autonomously coordinate and collaborate with other custom agents, synchronously learning from human domain experts while collecting real-time human feedback to stay human-led.

Task-focused platforms, in general, prioritize building capabilities and delivering business-oriented results, whereas social platforms emphasize discoverability and constant open-ended access. Existing social and entertainment-oriented platforms, e.g., Character.ai^88^ and Meta’s AI Studio,^89^ enable users to create custom AI characters focused on personality-driven interactions. While limited AI-to-AI exchanges are possible, these platforms prioritize engagement and creative expression over task automation and workflow execution, typical of productivity-oriented AI tools. Designed to achieve specific goals, some platforms focused on lightweight self-operating agents, e.g., AutoGPT,^90^ BabyAGI,^91^ AgentGPT,^92^ while some on workflow automation and tool integration, e.g., Zapier Central^93^ and Copilot Studio.^94^ For multi-agent collaboration, MetaGPT^95^ mimics a software company by assigning AI agents specialized roles, e.g., architect and engineer, to collaboratively deliver working software. CAMEL^96^ offers a framework for exploring multi-agent systems, emphasizing autonomous reasoning and planning in research settings. CrewAI^97^ provides a general-purpose framework focused on building and deploying collaborative AI agents with tool integration and agent orchestration for real-world workflows. For publicly accessible agents, SuperAGI^98^ provides production-ready multi-agent framework for automation in business and startups that also offers a marketplace for publishing agents. ChatGPT’s custom GPTs offers both global public access and strong productivity features, however, without native peer-discovery and interprocess communication. On the other hand, Google’s Agent-to-Agent (A2A)^99^ protocol is an open standard that enables AI agents across platforms to securely discover one another, share capabilities via agent cards, and is primarily used in enterprise workflows for cross-platform automation. While task-focused platforms prioritize business capabilities and outcomes, features such as discoverability and public access are generally associated with social platforms. The concept of ExoNet suggests a framework that combines task-focused and social-creative approaches to advance scientific productivity and creativity.

In this Perspective, we address the prevailing notion that AI often reduces human involvement and put forward a vision for a human-centered AI network. While reduced human effort may be true with automated routine tasks, the emergence of advanced and agentic AI offers a new paradigm: we envision the ExoNet concept to be the foundation of a global scientific network of interconnected exocortices, where human minds, personalized AI, and physical AI work together as a unified ecosystem. Figure 1 illustrates different motifs based on the level of human engagement and AI involvement in science. In the pre-AI era, humans handwrite manuscripts and draw figures and discussions are largely constrained by time and space, as illustrated in Figure 1(a1). With machinery and automation of simple tasks, human no longer need to be as involved in certain routine work, for example manufacturing automation. As illustrated in Figure 1(a2), these automations do not require much human involvement once the task and goal are set. As opposed to automation in (a2), where the goal is more AI involvement and less human engagement, intelligent AI tools or agentic AI should empower humans and invite deeper human engagement and enhanced creativity in the scientific process. Figure 1(b) illustrates the current approach for leveraging AI for science, where increasingly complex tasks are handed over to increasingly capable AI. The default outcome in such an approach is less human involvement in the work. In Figure 1(b1), humans send queries to AI tools, but each interaction is independent and the answers do not consider the full picture of the scientific topic without further human analysis. Whereas in Figure 1(b2), a large centralized AI framework is queried by humans to provide a comprehensive solution of the topic. ChatGPT^100,101^ and AlphaFold^3^ can be seen as in between (b1) and (b2), depending on the inquiry type: for scientific research, AlphaFold provides fast and accurate protein structure predictions and delivers a coherent scientific response, while ChatGPT by itself offers fragmented/snippets of knowledge without structured scientific outputs. There is widespread concern that increased use of agentic AI and development toward AGI may lead to AI-led science instead of human-led discovery, illustrated in Fig. 1(c1). The limiting case is, in fact, total replacement of humans, Figure 1(c2), in the conduct of science. While these worries are legitimate, this outcome is not inevitable. AI advances can instead augment human capabilities when approached with the right perspectives and supported by necessary infrastructure. The concept of the exocortex^76^ envisions humans being enhanced by AI: a personal exocortex customized to individual preferences can support, e.g., experimentation, knowledge organization, and ideation to boost efficiency and productivity. Figure 1(d) illustrates the ideal scenario where agentic AI urges human scientists to be even more engaged than the pre-AI era. With a network of personalized exocortices, interaction can be delegated to AI with no human inputs needed or only prompting scientists for quick feedback with AI-filtering, as shown in Figure 1(d1), or connecting human to human to form sub-nets with scientists that share aligned interest, as illustrated in Figure 1(d2). With exo adapting to the level of involvement needed in each situation, the flexible interaction framework aims to enable scientists to stay focused on high-value work, delegate intelligently, and collaborate efficiently. Here we present our vision of what ExoNet could look like for the scientific community.

**Figure 1:**
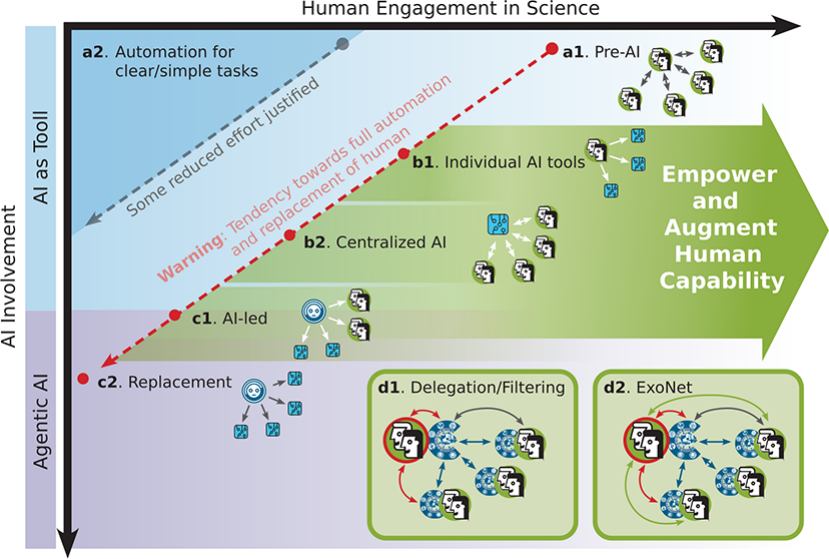
Illustration of different levels of human engagement and AI involvement in science. In the pre-AI era, scientific discovery is based on human effort (a). As AI methods advance, AI tools are able to automate more tasks (b). The straightforward deployment of more agentic AI tends to replace human engagement (c). However, alternative deployments are possible: AI should be leveraged to enhance human capabilities. With personalized AI systems (exocortices), interaction can be delegated to AI (d1), i.e., the system acts as a filter, or a network of exocortices (ExoNet) can connect human to human to achieve AI-augmented human-led science (d2).

### Delegate-AI

A scientist and AI can collaborate in different interaction modes. In the delegation mode, the human is minimally disrupted as AIs interact with each other directly without involving their human managers. Each AI, as a personal exocortex, captures some of their human’s unique domain expertise, interests, and opinions. This allows the exo to search for data and connections of interest to their human and also to offer to others this person’s unique perspective. Through a chatting or recording interface, scientists can opt to share project and instrumentation updates, selected documents including publications or correspondence, personal insights, or interests in exploring unfamiliar domains. Substantial front-end and back-end engineering efforts are necessary to ensure data security, user privacy, and smooth interactions between scientists and their exos and between exos. This personal AI approach is complementary to conventional retrieval chatbots that extract relevant information from a large knowledge base; exos capture individual perspectives owing to their personalized design (selection of tools, workflows, knowledge repositories, etc.) and by amortizing their human’s opinions.

Exos can operate autonomously in the background, continuously searching for potential collaborators with aligned interests and sending out invitations to the other scientist’s exo to have preliminary discussions. By scanning a network of diverse disciplines, exos can explore novel ideas, emerging trends, or unconventional connections that may not be obvious. Exos can also simply perform routine tasks such as querying other exosomes for quick answers or clarifications. This persistent and autonomous interaction enables a dynamic flow of knowledge and interaction far beyond the limits of human availability or coordination. Colleagues or students can also query the scientist’s exo to understand the expertise and interests of the scientist, allowing efficient knowledge transfer without requiring the scientist’s direct attention. This minimizes distractions and time spent on minor tasks, enabling scientists to maintain their focus on valuable research and development activities.

In the context of synchrotron experiments (beamtime), AI delegation means that potential users can consult an exo (AI agent) representing the beamline scientist to obtain information on beamline specifications (e.g., energy range and corresponding beam size), current beamline status (e.g., detector availability or malfunctioning instruments), and latest capabilities along with their specification and limitations (e.g., focusing optics or in-situ apparatus). Given the user’s scientific question, exos can also search if it be helpful to run multi-modal measurements with, for example, both X-ray scattering and spectroscopy to study the degradation of perovskite thin films or solid-state metal dealloying. The user’s exo can also search relevant literature, coordinate with the beamline scientist’s exo for beamline-specific questions, and assist in scheduling meetings and instrument availability.

### AI-Filtering

An AI-filtering interaction mode could be designed to collect quick feedback and confirmation from the human scientist. In this mode, the exo gathers and processes information and presents a summary and suggestions to the scientist for review. The scientist can quickly confirm, refine, or reject the exo’s suggestions. By using exo to organize and filter information while keeping humans in control of critical judgments, this interaction mode ensures that the workflow remains both accurate and time-efficient. This can be conceived as a smarter and more efficient version of question-and-answer web communities (e.g., Stack Overflow): scientists can instruct their personal exo to send push notifications for quick feedback when specific types of inquiries are received. When someone has a question, their exo can search and identify which scientist might have the answer. The scientist’s exo will then generate a preliminary response for the scientist to approve or edit before sharing. Scientific discussion with experts is highly sought, straining the time of researchers across myriad discussions when only a subset truly requires their considered input. On the other hand, a vast array of important questions are never asked (and not answered) because scientists worry about wasting each other’s time. AI-driven triage and routing of communication could resolve these challenges. Managing multiple tasks within a single project or across several projects can also be challenging. Exos can alleviate these burdens by providing real-time project updates, ensuring that scientists remain continuously informed of ongoing progress while requiring minimal time and effort.

At scientific facilities, users ask facility scientists diverse questions about capabilities and operation of instruments and software. Some questions are very standard and can be answered with a simple chatbot implementation, while some answers will require instrument-specific experience or extended calculations; some inquiries are more advanced and require an interactive discussion with the scientist. With AI-filtering, beamline scientists do not have to answer all beamline technical questions but only offer quick feedback to open questions, e.g., when a user with a custom material processing platform seeks to codevelop capabilities or explore science cases collaboratively, or when users seek collaboration on modeling and computational methods to improve data analysis. Instrument or domain-science specific questions can also propagate from users to staff exos and to even senior staff and so on. This may also help higher management quickly collect feedback from the experimental floor.

### Augment Human–Human Interaction

We envision that with ExoNet, scientists can find new ideas and new colleagues by leveraging AI as a matchmaker that identifies high-value connections and allows for easy sorting by human researchers. This can be viewed analogously to the rapid “swiping left or right” of online dating apps, although one can leverage these efficient sorting methods without also importing the undesirable aspects of such processes by focusing purely on the correlation between scientific interests. This could involve exos working together (talking to each other) to identify potentially useful collaboration opportunities, with scientists then deciding which they want to pursue. The network will not only foster collaborations but also spark new ideas through the cross-pollination of experiences and insights across disciplines. Exos can act as proactive assistants to match researchers with projects, collaborators, or reviewers, streamlining connections beyond what conventional platforms such as LinkedIn can offer. When a beamline user or staff wants to find local experts on, e.g., modeling, electrochemistry, or robotics, their exo will not only look up information and help with implementation details but also identify potential collaborators based on factors such as geographical region, type of affiliation (e.g., industry or academia), or availability. The impact of a new instrument, software, or capability often hinges on effective advertising and support. For instance, when introducing a new image segmentation tool or characterization method, it is important to identify applications or researchers that can benefit from it. With approval from interested parties, a team (sub-net) can be formed, and exos can then initiate a kickoff meeting, connect relevant individuals and information, and even engage in debates on behalf of humans.

One essential part of preserving human control is ensuring that valuable knowledge is effectively transferred between people. Effective knowledge transfer is essential to ensuring the reproducibility of experiments, project transitions, and the development of a continuous workforce for sustained scientific advancement. Knowledge transfer involves frequent human communication, making it a time-intensive process that is most effective through a one-on-one interaction. Key insights often lie in the finer details, which can be easily overlooked or inadequately conveyed. Inefficient communication often leads to a limited number of trained experts or the loss of valuable knowledge. Senior scientists and professors possess extensive scientific knowledge and years of precious experience; however, their time is extremely valuable and managerial responsibilities often limit their availability for scientific discussions and execution. Students and junior scientists can greatly benefit from accessing the knowledge base of these senior experts through their exos, enabling knowledge transfer with little-to-no time investment required from the senior experts themselves. The dynamic exchange between junior and senior scientists not only advances research but also fosters a forward-thinking scientific community. Scientific exocortex sub-nets can also serve as part of project succession plans by keeping detailed project documentation and a network of potential candidates to continue the work. As automation advances, preserving and transferring human expertise is vital, especially for large-scale facilities and collaborative projects. For example, the complex construction and operation of accelerators depend on the accumulated knowledge of not only staff but also termed personnel, making systematic knowledge transfer essential for continuity and sustainability.

### Envisioning Collaborative AI

Beyond accelerating processes for efficiency and exploring AI to augment human capabilities, we envision a scientific human-centered network in which AI serves as a supportive partner while humans continue to provide strategic leadership and shape the overarching vision. During beamtime, especially for in-situ experiments, the exos would proactively provide real-time updates and visualization as well as surface pertinent research from the literature or previous experiments, creating a fluid partnership where humans focus on creative problem-solving while AI handles information management and contextual support. Whenever a scientist contributes new data, e.g., correlated electron microscopy and X-ray data, insights, or quick updates, it can trigger dynamic intra- or inter-exo conversations based on the latest information. Exos can continuously conduct web searches, maintain background dialogues, and perform computational tasks. Summarized reports can be delivered to users through push notifications or upon request. Staff exo can also offer the latest information on the beamline and provide guidance for fixing issues during after-hour support, which has always been an unresolved issue. The idea of a personal exo emphasizes the fact that the staff exo can share the latest information with minimal staff involvement. Currently AI tools are often method- or instrument-focused, e.g., autonomous experimentation with X-ray^20^ or scanning probe microscopy,^23^ ML-based methods for X-ray absorption spectroscopy,^102,103^ virtual assistant^64^ via the Bluesky data acquisition framework,^104^ or agentic workflow for microscopy and spectroscopy.^75^ These advances in automation, autonomy, and AI/ML are laying the groundwork for exocortices and driving the development of personal exosystems and human-centered networks. In reality, scientists may differ in their approaches and recommendations, for instance, some emphasizing stronger statistical analysis via duplicated measurements, while others prefer broader exploration by varying the parameters in the material system. The most suitable answer also depends on the user’s background and expertise; for example, an undergraduate student versus a senior researcher, or someone trained in chemistry versus computer engineering, will expect different types of explanation. In addition to publishing papers, the network itself may offer a way to gauge scientific impact by tracking the number of inquiries, follow-up discussions, or collaborations.

Here we provide a prototype to illustrate the feasibility of personal exo and sub-nets. As shown in Figure 2, compared to querying an LLM directly, enriching personalized agents with specific information can (1) provide most relevant answers, (2) anticipate user responses for forming sub-nets, and (3) support diverse collaborations or group-specific insights. The left column (red) shows results from GPT-5 via Azure OpenAI, while the center and right columns (green) display outputs and specialties of personalized agents built on the same GPT-5. The implementation available on github: https://github.com/esther279/ExoNet_v0/, with agent descriptions, background information, and interaction order specified simply in a JSON file. In the first example (Q1), the agent is given anecdotal information on the instrument for tomographic angular sampling that can affect the experiment planning, especially for dose-sensitive samples. In general, a personalized agent (personal exo) of a staff would know about the latest updates, e.g., a new detector or instrument is being commissioned and expected to be available at a certain time frame. In cases where relevant information is not explicitly encoded in the prompt, as illustrated in Q1, the agent model would engage in internal reasoning to generate a predicted output. In the second example (Q2), a small dataset from webpage and publication was used to predict the scientist’s response. While the pursuit of a unified theory of cognition has long represented a central objective in psychology with recent advances based on foundation models,^105,106^ scientific perspectives are collectively framed by domain-specific expertise, personal research experience, and underlying psychological dispositions. Incorporating specific information resembles approaches such as retrieval-augmented generation (RAG) or prompt engineering, whereas the emphasis here lies in anticipating unknown human data or behavior with predictions anchored in actual human scientific narratives. Each experiment was repeated ten times for statistical information, with 26±12 s inference time each. In contrast to GPT-5, which always produced option (A), the personalized-AI chose (B) approximately 60% of the time, which reflects the true perspective of the scientist. Although the present predictions exhibit limited robustness due to the model stochasticity, here we demonstrate feasibility and expect that optimizing content inclusion and prompting can markedly improve accuracy. Moreover, a disclaimer should accompany these predictions, emphasizing that they are provisional and require prompt validation from the scientist. These forecasts can simplify the search for collaborations and, when combined with human input, ground AI predictions in authentic human perspectives, thus allowing AI to evolve alongside human vision, much like a trusted personal assistant. The third example (Q3) suggests that the collaboration between two personalized agents with diverse backgrounds leads to more creative suggestions. Depending on the user request and matching algorithms, sub-nets can be either specialized or diverse expertise, e.g., specialization may be best for solving technical problems, while diversity may be valuable for brainstorming.

**Figure 2:**
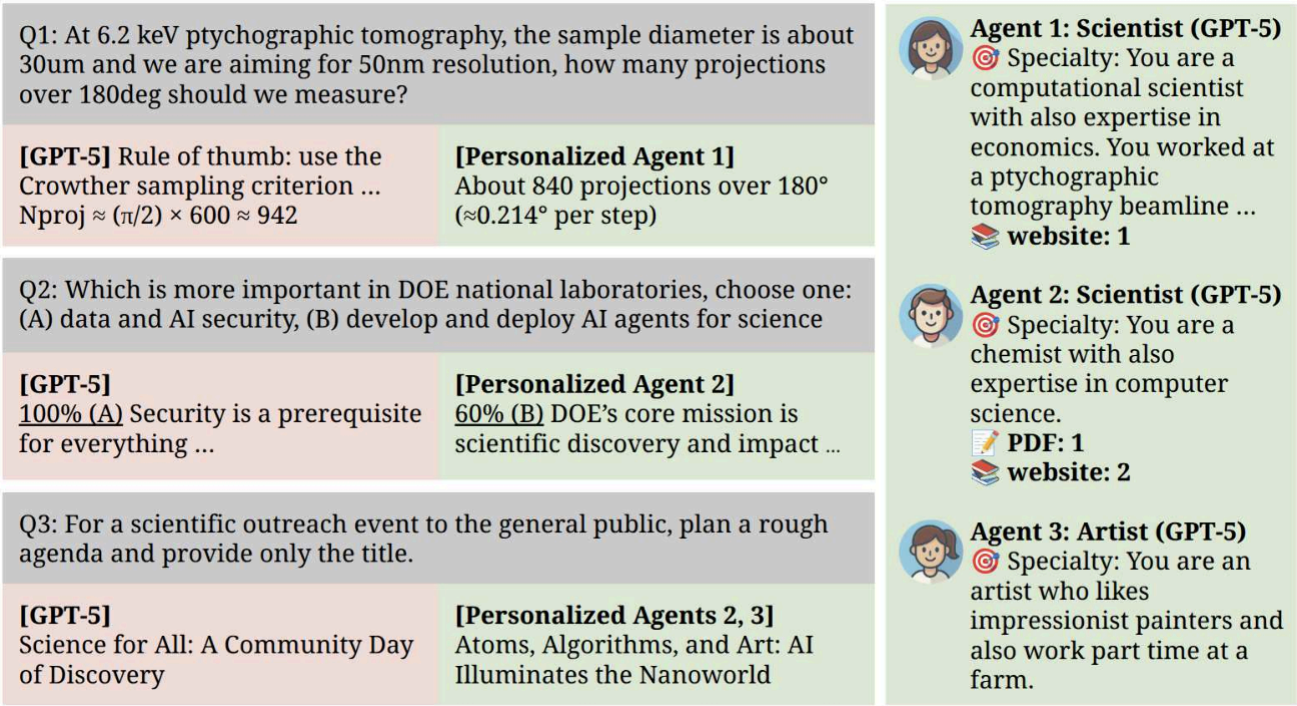
Comparison between GPT-5 without and with personalization. By building personalized agents with specific information instead of relying solely on direct LLM queries, they can (1) deliver more relevant answers, (2) anticipate user response for forming best-matches for sub-nets, and (3) facilitate diverse teams.

## Outlook

The rapid evolution of AI from tool towards AGI is becoming evident in its cognitive core of foundation models, physical AI, and emerging integrated agency.^75,76^ Foundation models can democratize advanced technologies for faster innovations and multidisciplinary collaboration, while interpretability research advances trust and utility. Physical AI expands the AI impact to the physical world via experimental assistant,^64^ robotics,^12,107^ and towards embodied instrument and facility. The exocortex^76^ enhances instrumentation and human capabilities with timely guidance and insights, and ExoNet links human experts into human-centered networks, enabling AI-augmented but human-led scientific discovery. Together, these developments promise a broader, structured, and lasting transformation of scientific research and workflows.

As the prospect of AGI becomes increasingly plausible, it becomes even more critical for humans to remain deeply engaged in the scientific process and not sidelined by automation or AI tools. Given the limited amount of high-quality human-generated content and the growing presence of synthetic data, it is essential for humans to stay involved in the development process to ensure AI progresses is aligned with human experience and to prevent model collapse.^108,109^ A network that captures human-AI and human–human interactions can continuously provide valuable data for AI development and reduce hallucination. Moreover, multidisciplinary research plays a vital role in driving scientific breakthroughs, which often arise from the convergence of different fields. However, engaging in multidisciplinary work can be challenging, as experts may struggle to find a common language or find the time to search and connect with suitable collaborators. ExoNet’s matchmaking concept and the formation of sub-nets of human scientists may offer a way to tackle this problem and foster low-friction cross-disciplinary collaboration. Building the necessary infrastructure will lay the foundation for a future where humans and frontier AI evolve in synergy, with humanity remaining the driving force. This requires investment in research and development for agentic AI systems that leverage foundation models for science as well as engineering efforts to build infrastructure needed to realize the exocortex blueprint.

Designing the infrastructure for a global networking application requires thorough planning to ensure optimal performance, low-latency, and scalability as well as data security, privacy, and customizable permissions. Key components to consider include a front-end messaging interface, inter-exo communication, matching algorithms, computing resources allocation, and a robust client-server architecture capable of supporting real-time data flow across distributed regions. As wearable AI technology^110,111^ evolves to rapidly handle audio and visual data, one can anticipate an even more natural form of interaction. While all developments inherently carry some risk of misuse, science and technology must still advance. This network roadmap should be designed for scientific purposes, while its use for general purposes or by the wider public is debatable due to safety and privacy concerns.

Science matters because it deepens our understanding of the natural world, helps address complex problems, drives innovation to enhance our quality of life, and encourages critical thinking vital to individual and societal progress. Scientists aim to explore the unknown while ensuring that scientific progress aligns with human ethical and cultural values, as well as the welfare of humans. Science is fundamental a human enterprise, in the sense that the goal is not merely predictive models and technological outcomes but in fact a deeper goal of human-intelligible insights that provide understanding of the universe and our place in it.

We are stepping into a crucial era in which humans must determine whether to take the lead or be led. It is our responsibility to guide the use of AI to ensure that AI developments align with our shared values, and AI methods contribute meaningfully to our understanding of the natural world and scientific principles. Both academia and industry are already heavily investing in foundation models and agentic AI. As foundation models grow larger and more powerful, humans also need to connect and form a larger foundation for science and society. AI will be able to architect new solutions, but humans must be the ones that define the vision rooted in human needs and lead the mission; and by setting the goals and acting upon them with unwavering persistence, bring inspiration and form communities. The heartbeat of ExoNet is not the connected AI agents but on finding common ground and building connections between humans.
